# Barriers to HIV pre-exposure prophylaxis among African, Caribbean and Black men in Toronto, Canada

**DOI:** 10.1371/journal.pone.0213740

**Published:** 2019-03-29

**Authors:** Alice Zhabokritsky, LaRon E. Nelson, Wangari Tharao, Winston Husbands, Ting Sa, Nanhua Zhang, Jamie Thomas-Pavanel, Shamara Baidoobonso, Rupert Kaul

**Affiliations:** 1 University of Toronto, Department of Medicine, Toronto, Canada; 2 St. Michael’s Hospital, Li Ka Shing Knowledge Institute, Center for Urban Health Solutions, Toronto, Canada; 3 University of Rochester School of Nursing, Rochester, NY, United States of America; 4 Women’s Health in Women’s Hands, Toronto, Canada; 5 Ontario HIV Treatment Network, Toronto, Canada; 6 Cincinnati Children’s Hospital Medical Center, Department of Biostatistics and Epidemiology, Cincinnati, OH, United States of America; 7 Women’s College Research Institute, Women’s College Hospital, Toronto, Canada; 8 Chief Public Health Office, Charlottetown, Prince Edward Island, Canada; 9 University of Toronto, Departments of Medicine and Immunology, Toronto, Canada; Centers for Disease Control and Prevention, UNITED STATES

## Abstract

**Introduction:**

Single-tablet combination emtricitabine/tenofovir is highly effective as HIV pre-exposure prophylaxis (PrEP). Scale-up efforts have targeted men who have sex with men (MSM), but patterns of racial disparities in PrEP use have begun to emerge. African, Caribbean and Black (ACB) communities in Canada and USA are also disproportionately affected by HIV, and there is lack of guidance regarding PrEP implementation in this priority population.

**Methods:**

ACB men from Toronto, Canada were recruited in community settings by peers. Participants completed a detailed socio-behavioural questionnaire. Biological samples were collected and tested for sexually transmitted infections. Willingness to accept PrEP was assessed in relation to actual and self-perceived risk of acquiring HIV, as well as demographic and behavioural variables.

**Results:**

424 ACB men were included in the analysis. ACB MSM were more likely to accept PrEP than ACB men only reporting sex with women (MSW; 50.0% vs. 23.6%). The most common reasons for PrEP non-acceptance were concerns regarding side-effects and low self-perceived risk. PrEP acceptance was lowest among younger men (12.5%) and those born in Canada (15.2%). Men with a high self-perceived HIV risk were more likely to accept PrEP (41.3% vs. 22.7% of men with a low self-perceived risk), but only 25.4% of men who were defined as being at high-risk, self-identified themselves as such.

**Conclusions:**

Most ACB MSW were unlikely to accept PrEP, largely due to low self-perceived HIV risk, but PrEP acceptance among ACB MSM was similar to other contemporaneous Toronto MSM communities. PrEP acceptance was particularly low among younger ACB men and those born in Canada. Tailored strategies will be needed to effectively implement PrEP in Toronto ACB communities.

## Introduction

HIV remains an important public health issue, with an estimated 1.8 million new infections worldwide in 2016 [1]. Over the last several decades there have been substantial advances in the fields of HIV prevention and treatment. The development of single-tablet combination antiretroviral regimens has transformed the clinical management of people living with HIV, resulting in excellent virologic suppression, a near normal life expectancy [2], and the virtual elimination of secondary sexual HIV transmission [3,4]. Dual-agent regimens also reduce HIV acquisition risk by over 90% among uninfected, at-risk individuals if taken as pre-exposure prophylaxis (PrEP) [5,6], although efficacy is very dependent on the degree of medication compliance. In February 2016, Health Canada approved the use of a single-tablet combination tenofovir/emtricitabine (Truvada) as PrEP, and the first Canadian guidelines for PrEP have since been published [7]. Several provinces across Canada, including Ontario, have added PrEP to their provincial drug formulary of publicly funded drugs, significantly improving accessibility.

Despite these advances in HIV treatment and prevention, the number of new HIV diagnoses in Canada has been relatively stable over the last two decades [8], with Ontario accounting for the highest number and proportion of reported cases (n = 881/2344 in 2016, 37.6%). Just under half of these infections were among men who have sex with men (MSM), and a third acquired through heterosexual transmission; in both contexts, members of African, Caribbean and other Black (ACB) communities were disproportionately affected. Indeed, while representing just 3.5% of Canada’s population [9], ACB communities accounted for 21.9% of new diagnoses across Canada in 2016 [8]. To date there is little information available regarding the patterns of healthcare access and the acceptability of PrEP in this population, particularly among ACB men.

Despite the efficacy of biomedical HIV interventions, there may be challenges to their implementation in different communities and populations. Current Canadian guidelines recommend PrEP for individuals at high risk of HIV, including those in serodiscordant relationships, and have been tailored to screen MSM based on recent sexual behavior and sexually transmitted infections (STIs) [7]. In persons from other priority populations, access to PrEP is currently more reliant on self-perceived HIV risk, but there can be significant discrepancies between perceived and actual HIV risk [10]. In addition, while PrEP uptake and knowledge are improving in MSM communities [11,12], awareness is lower among ACB men [13–15]. In order to design public health strategies to ensure equity in the implementation of PrEP in populations at high-risk for seroconversion, we recruited a community-based sample of ACB men from Toronto, Canada and assessed the relationship between biosocial factors and acceptability of PrEP, looking at overall rate of acceptance and barriers to PrEP uptake.

## Methods

### Study design and data collection

Ethics approval for this study was obtained through HIV Research Ethics Board at the University of Toronto, and full details of the study design, recruitment and data collection have been previously described (*Nelson LE*, *Tharao W*, *Husbands W*, *Sa T*, *Zhang N*, *Kushwaha S*, *Kaul R*. *The epidemiology of HIV and other sexually transmitted infections in African*, *Caribbean and Black men in Toronto*, *Ontario; unpublished manuscript*) [16]. In brief, a cross-sectional descriptive epidemiological study enrolled ACB men from Toronto, Canada between 2011 and 2013. Men were recruited by a team of formally trained ACB peer recruiters using a variety of engagement strategies, including street interception, social networks, as well as venue and event-based approaches. Events and community organizations known to be popular with ACB communities in Toronto were selected for recruitment (i.e. Afrofest, Caribana, Black Daddies Club and Jamaican-Canadian Association). A community-based research approach was used to engage ACB men who may or may not be engaged in healthcare, allowing for better representation of ACB communities.

Survey completion and sample collection were performed at one of three designated community health centres. Survey data were collected using an audio computer-assisted self-interview (ACASI), capturing social and behavioural variables, including age, education, sexual partnering and region of birth. Biological specimens included blood, urine and swabs from the anus and penis. Serum enzyme immuno-assays were performed for HIV, herpes simplex virus (HSV)-2 and syphilis. All reactive HIV results were confirmed by Western blot. A nucleic acid amplification test was used to detect *Neisseria gonorrhoeae* and *Chlamydia trachomatis* infection (Becton Dickinson ProbeTec nucleic acid amplification and detection system). High-risk oncological human papillomavirus (HPV) subtypes were detected using self-administered anal and penile swabs by polymerase chain reaction (PCR; Roche linear array).

### Study measures

Access to healthcare was assessed by asking participants if they have a family physician and whether they have visited their primary care physician in the last 6 months. They were also asked whether they visited any other type of healthcare provider (i.e., walk-in clinic, emergency room, nurse practitioner) in the last 6 months. As was done in previously published studies [17], knowledge about HIV and other STIs was assessed by using 17 true and false statements and asking participants to state whether they agree or disagree (S1 Table). Degree of knowledge was calculated by summing the total number of correct answers, and analyzed as a continuous variable. HIV stigma was measured by using 10 statements related to exposure to, and acceptance of, people living with HIV in social and professional environments, as well as by views on disclosure of HIV status to family members and sexual partners. Questions were based on two validated indicators of HIV related stigma by UNAIDS/WHO and USAID [18,19], although combined use of the indicators has not been validated in other studies. HIV stigma was quantified by summing the number of answers that corresponded with stigmatizing views, and analyzed as a continuous variable.

Self-perceived risk for acquiring HIV was assessed by asking participants how likely they thought they were to become infected with HIV. Those responding with ‘certain’, ‘highly likely’ or ‘somewhat likely’ were categorized as high self-perceived risk and those responding with ‘somewhat unlikely’, ‘highly unlikely’ or ‘not at all’ were categorized as low self-perceived risk. Actual risk of HIV acquisition was defined as ‘high’ if a participant either (1) reported condom-less sex with an HIV-positive partner in the last 6 months; or (2) reported condom-less sex with a partner of unknown HIV status in the last 6 months *and* had a lab-confirmed STI (HSV-2, syphilis, chlamydia, gonorrhea or high-risk penile/anal HPV).

The concept of HIV PrEP was introduced in a statement describing the evidence for its protection against acquiring HIV based on the iPrEx study [20], and participants were asked how likely they were to take PrEP: “*Recently*, *a study among HIV-uninfected persons given anti-HIV drugs on a regular basis reported some protection (40–70%) from becoming infected with HIV; this is called Pre-Exposure Prophylaxis (PrEP)*. Thinking about your risk of HIV, how likely would you be to take PrEP?”. Those unlikely to accept PrEP were asked to elaborate and were given the options of either providing a free text explanation, or of selecting any one of: “low self-perceived risk for acquiring HIV”, “concern regarding side effects”, “pill burden” or “belief that PrEP is ineffective”.

### Statistical analysis

All analyses were conducted using SPSS v. 24.0 (IBM Corp., Armonk, NY, USA). Age was summarized using mean, standard deviation and proportions, and remaining demographic data were summarized using proportions. Logistic regression models were used to assess which demographic and other characteristics were associated with PrEP acceptance. Odds ratios (OR), 95% confidence intervals (CI) and *p* values are reported. Variables affecting participants’ decision to decline PrEP were summarized using proportions. Association between perceived and actual risk for acquiring HIV was assessed using the Pearson Chi-Square test. Logistic regression was used to assess whether the accuracy of the self-assessment of risk was associated with a participant’s level of education, degree of knowledge about STIs or stigma against HIV.

## Results

### Study population

A total of 486 ACB men took part in the larger study: participants who were seropositive for HIV (*n* = 46) and those who reported never having been sexually active (*n* = 16) were excluded from analysis, giving a final sample size of 424 participants (Table 1). The mean participant age was 35 years (*SD* = 13.9 years). Most men were born outside of Canada (*n* = 259; 61.1%), most commonly in countries in the Caribbean (*n* = 135; 52.1% of those born outside of Canada) or Africa (*n* = 107; 41.3%). Most participants (*n* = 382; 90.1%) reported having only ever had sex with women (MSW), with a smaller proportion (*n* = 42; 9.9%) reporting sex with another man (MSM). Almost half of participants had completed some post-secondary education (*n* = 182; 42.9%), with the remainder having completed a high school diploma or less. Most (71.2%) reported having a family physician, and half of these had visited their primary healthcare provider within the last 6 months (*n* = 154; 51% or 37% of total study population). A larger proportion of men had visited any healthcare provider in the last 6 months (n = 327; 77.1%).

**Table 1:** Demographic characteristics of study participants.

Characteristic	Total (n = 424) no. (%)
Age–years ± SD	35 ± 13.9
15–24	136 (32.1%)
25–34	87 (20.5%)
35–44	84 (19.8%)
45–54	80 (18.9%)
55+	37 (8.7%)
Region of birth	
Canadian born	165 (38.9%)
Born outside of Canada	259 (61.1%)
Africa	107 (25.2%)
Caribbean	135 (31.8%)
Other	17 (4.0%)
Sexual partnering	
MSM	42 (9.9%)
MSW	382 (90.1%)
Level of education	
Some/completed secondary school	242 (57.1%)
Some/completed college or university	182 (42.9%)
Has a family doctor	302 (71.2%)
Visited family doctor in the last 6 months	154 (51.0% of those with a family doctor, 37% of all study participants)
Visited a healthcare provider in last 6 months	327 (77.1%)

*SD*: Standard deviation

### PrEP acceptance

The majority of participants reported being unlikely to accept PrEP (73.8%; Table 2). MSM were more likely to accept PrEP than MSW (50% vs. 23.6%, OR = 2.04; 95% CI 1.00–4.16; *p* = 0.051; Tables 2 and 3). Men in the younger age groups were less likely to accept PrEP than their peers (12.5% acceptance rate among 15–24 year-olds, and 26.4% among 25–34 year-olds), with the average age of those likely to accept PrEP being 40 years vs. 34 years for those unlikely to accept PrEP. Participants born in Canada were also less likely to accept PrEP (15.2%) than those born outside of Canada (33.2%), with no significant difference in PrEP acceptance between Caribbean-born and African-born men (OR = 1.06; 95% CI: 0.62–1.81; *p* = 0.839). PrEP acceptance was not associated with a higher level of education (OR = 0.85; 95% CI: 0.51–1.40; *p* = 0.520) or the degree of HIV stigma (OR = 0.94; 95% CI: 0.80–1.10; *p* = 0.416). Degree of knowledge about HIV and other STIs had minimal (although statistically significant) impact on decision regarding PrEP acceptance, with higher knowledge being associated with higher acceptance (OR = 1.10; 95% CI: 1.02–1.19; *p* = 0.014). Having access to a family physician was not associated with PrEP acceptance (OR = 0.75; 95% CI: 0.42–1.37; *p* = 0.354), nor did visiting the family physician or another healthcare provider in the last 6 months (OR = 0.98; 95% CI: 0.53–0.1.83; *p* = 0.956 and OR = 1.11; 95% CI: 0.59–2.08; *p* = 0.755).

**Table 2:** Crude logistic regression analysis of variables associated with participants accepting PrEP.

Characteristic	Accept PrEP (n = 111, 26.2%) no. (%)	Decline PrEP (n = 313, 73.8%) no. (%)	OR (95% CI)	*p* value
Age–years			1.03 (1.02–1.05)	0.000
15–24	17 (12.5%)	119 (87.5%)		
25–34	23 (26.4%)	64 (73.6%)		
35–44	31 (36.9%)	53 (63.1%)		
45–54	28 (35%)	52 (65%)		
55+	12 (32.4%)	25 (67.6%)		
Region of birth			0.36 (0.22–0.60)	0.000
Canadian born^1^	25 (15.2%)	140 (84.8%)		
Born outside of Canada	86 (33.2%)	173 (66.8%)		
Sexual partnering			3.24 (1.66–6.21)	0.000
MSM^1^	21 (50%)	21 (50%)		
MSW	90 (23.6%)	292 (76.4%)		
Education			1.30 (0.84–2.01)	0.233
≥ College or university^1^	53 (29.1%)	129 (70.9%)		
≤ Secondary school	58 (24%)	184 (76%)		
Access to family doctor			0.84 (0.52–1.34)	0.455
Yes^1^	76 (25.2%)	226 (74.8%)		
No	35 (28.7%)	87 (71.3%)		
Visited family doctor^2^			1.21 (0.78–1.90)	0.398
Yes^1^	44 (28.6%)	110 (71.4%)		
No	67 (24.8%)	203 (75.2%)		
Visited any healthcare provider^2^			1.37 (0.80–2.36)	0.249
Yes^1^	90 (27.5%)	237 (72.5%)		
No	21 (21.6%)	76 (78.4%)		
Degree of stigma against HIV^3^			0.91 (0.79–1.06)	0.220
Knowledge about STIs^3^			1.10 (1.03–1.27)	0.006
Perceived risk of acquiring HIV			2.40 (1.44–4.00)	0.001
High^1^	33 (41.3%)	47 (58.8%)		
Low	78 (22.7%)	266 (77.3%)		
Actual risk of acquiring HIV			1.69 (0.98–2.91)	0.060
High^1^	25 (35.2%)	46 (64.8%)		
Low	86 (24.4%)	267 (75.6%)		

^1^Referent group

^2^In the last 6 months

^3^ Continuous variable from low to high

*OR*: Odds Ratio, 95% *CI*: 95% Confidence Intervals, *PrEP*: Pre-exposure prophylaxis, *MSM*: Men who have sex with men, *MSW*: Men who have sex with women only, *STI*: Sexually transmitted infection, *≤*: Less than or equal to, *≥*: More than or equal to

**Table 3:** Adjusted logistic regression analysis of variables associated with participants accepting PrEP.

Characteristic	OR (95% CI)	*p* value
Age–years	1.03 (1.01–1.05)	0.009
Region of birth (Canadian born^1^ vs. other)	0.48 (0.27–0.85)	0.011
Sexual partnering (MSM^1^ vs. MSW)	2.04 (1.00–4.16)	0.051
Education (> Secondary school^1^ vs. less)	0.85 (0.51–1.40)	0.520
Access to family doctor (Yes^1^ vs. No)	0.75 (0.42–1.37)	0.354
Visited family doctor^2^ (Yes^1^ vs. No)	0.98 (0.53–1.83)	0.956
Visited any healthcare provider^2^ (Yes^1^ vs. No)	1.11 (0.59–2.08)	0.755
Degree of stigma against HIV^3^	0.94 (0.80–1.10)	0.416
Knowledge about STIs^3^	1.10 (1.02–1.19)	0.014
Perceived risk of acquiring HIV (High^1^ vs. Low)	2.04 (1.16–3.59)	0.014
Actual risk of acquiring HIV (High^1^ vs. Low)	1.48 (0.82–2.68)	0.192

^1^Referent group

^2^In the last 6 months

^3^ Continuous variable from low to high

*OR*: Odds Ratio, 95% *CI*: 95% Confidence Intervals, *MSM*: Men who have sex with men, *MSW*: Men who have sex with women only, *STI*: Sexually transmitted infection, *>*: More than

The most common reasons for non-acceptance of PrEP were concerns about PrEP side-effects (45%) and a low self-perceived risk for acquiring HIV (38%). A few participants reported inefficacy of PrEP (3%) and concerns around high pill burden (2%) as their reason for being unlikely to accept PrEP. Participants who selected “other” (12%) as their reason for declining PrEP generally also fitted into the “low self-perceived HIV risk” category.

### Perceived and actual risk for acquiring HIV

Overall, ACB men with high self-perceived risk for acquiring HIV were significantly more likely to accept PrEP compared to those with low self-perceived risk (OR = 2.04; 95% CI: 1.16–3.59; *p* = 0.014); this association was specific for ACB MSW (OR = 2.43; 95% CI: 1.36–4.33; *p* = 0.003), and self-perceived risk assessment among MSM was not associated with PrEP acceptance (OR = 1.00; 95% CI: 0.30–3.40; *p* = 1.000). While men with high actual risk for acquiring HIV were more likely to accept PrEP, this association was weaker and did not reach statistical significance (OR = 1.48; 95% CI: 0.82–2.68; *p* = 0.192), regardless of sexual partnering (OR = 0.73; 95% CI: 0.20–3.11; *p* = 0.726 –among MSM and OR = 1.80; 95% CI: 0.99–3.28; *p* = 0.054 –among MSW). In addition, only a quarter of men who were classified as being at high actual risk of acquiring HIV, self-identified themselves as such (Table 4), although most men classified as being at low actual HIV risk self-assessed their risk as low (82.4%). Again, discrepancies were noted between MSM and MSW: almost half of MSM (45.5%) who were classified as being at high actual risk of acquiring HIV self-identified as such, compared to just 21.7% of MSW. Overall, no statistically significant association was found between “perceived risk” and “actual risk”, whether stratified by sexual partnering or not (Table 4). There was no significant association between the accuracy of perceived risk (relative to “actual risk”) and the level of participant education (OR = 1.07; 95% CI: 0.69–1.65; *p* = 0.764), degree of knowledge about STIs (OR = 1.00; 95% CI: 0.94–1.06; *p* = 0.974) or stigma against HIV (OR = 0.90; 95% CI: 0.79–1.03; *p* = 0.117).

**Table 4:** Pearson Chi-Square test comparing perceived and actual risk of acquiring HIV.

	All participants		MSM		MSW	
Perceived risk	Low actual risk	High actual risk	Low actual risk	High actual risk	Low actual risk	High actual risk
Low	291 (82.4%)	53 (74.6%)	18 (58.1%)	6 (54.5%)	273 (84.8%)	47 (78.3%)
High	62 (17.6%)	18 (25.4%)	13 (41.9%)	5 (45.5%)	49 (15.2%)	13 (21.7%)
	X^2^(1, *N* = 424) = 2.34, *p* value = 0.126		X^2^(1, *N* = 42) = 0.04, *p* value = 0.840		X^2^(1, *N* = 382) = 1.55, *p* value = 0.214	

*OR*: Odds Ratio, 95% *CI*: 95% Confidence Intervals, *MSM*: Men who have sex with men, *MSW*: Men who have sex with women only

## Discussion

The daily administration of single-tablet combination emtricitabine/tenofovir as PrEP is an effective method to prevent HIV acquisition. MSM communities are most affected by the HIV epidemic in US and Canada, and the majority of PrEP implementation studies have focused on MSM [5,6,21]. However, ACB communities are also disproportionately affected by HIV [6], and much less is known about the acceptability of PrEP in this context, with no prior data from ACB men in Canada. In our community-based study, most ACB men reported being unlikely to use PrEP, although MSM within the ACB community had a similar rate of PrEP acceptance as other MSM in contemporaneous studies [22,23]. Self-perceived risk of acquiring HIV was a significant factor in MSW participants’ likelihood to accept PrEP, with those men who felt they were at high risk for acquiring HIV being more likely take PrEP, but the ability of ACB men to self-assess HIV risk appeared to be sub-optimal.

Previous studies of PrEP acceptability among MSM have also found that concerns around access, side effects and inefficacy were common barriers to acceptance [22–24]. Our study highlights several specific challenges that can be anticipated with rollout of PrEP among ACB men. ACB MSW were significantly less likely to accept PrEP than ACB MSM, suggesting that there are additional barriers to PrEP uptake in this population. Demographic factors that were associated with lower likelihood to accept PrEP included younger age and being born in Canada, for reasons that will need to be explored in future studies; however, potentially modifiable factors such as stigma towards people living with HIV and healthcare access were not associated with PrEP uptake. Taken together, our study highlights substantial heterogeneity in PrEP perceptions among ACB men, and suggests that targeting interventions towards specific subpopulations may be of benefit in conjunction with ongoing efforts to address the barriers that have been seen across studies, such as concerns about side effects.

One of the strongest predictors of PrEP acceptance among MSW was self-perceived risk for acquiring HIV. However, only a fifth of MSW who were identified to be at an actual high HIV risk (based on recent sexual patterning and STI diagnostics) self-identified as being at high-risk, irrespective of their level of education or knowledge about HIV/STI transmission, symptoms and treatment. Self-assessment of risk among MSM also aligned poorly with actual risk and did not seem to play an important role in PrEP acceptance. Therefore, objective risk assessment of ACB men may be a critical component of PrEP implementation. Furthermore, providers must be provided with effective risk assessment tools, since under-estimation of risk or lack of familiarity with PrEP among providers negatively impacts uptake of PrEP [25]. Validated screening tools are available to healthcare providers for assessment of risk in MSM [26], and our study highlights the importance of developing similar tools for other high-risk populations including ACB men; the development of tools that would facilitate self-assessments of risk might also be of benefit.

There were several limitations to this study. Most notably, participants were provided with the best available evidence for PrEP efficacy at the time of the study (40–70%) based on the iPrEx study [20], which was a modest protective effect compared to that seen in subsequent implementation studies with higher participant compliance (i.e., >85–100%) [5,6], and this low stated efficacy might have discouraged some participants from accepting PrEP. However, very few participants reported this as their reason for declining PrEP, making it unlikely to have significantly impacted our conclusions. Secondly, our assessment of actual HIV risk did not account for the viral load of HIV-positive partners, which we now know to play a critical role in transmission [3,4]. This might have contributed to an over-estimation of actual risk in some participants, and should be accounted for when assessing indications for PrEP use in clinical settings. Furthermore, the true HIV risk for participants that we deemed to be at a high “actual” risk, based on (1) condom-less sex with an HIV-infected partner or (2) condom-less sex with a partner of unknown status in the context of a lab-diagnosed STI, is unknown and is likely to be lower than that of MSM deemed to be high-risk based on criteria such as the HIRI-MSM index [26]. Finally, HIV stigma is a complex and multifaceted concept. In our questionnaire we only looked at a few HIV stigma domains, which might not have captured important aspects of stigma impacting ACB men in their decision to accept PrEP. Certainly, in MSM communities stigma has been shown to be an important barrier to PrEP uptake [27,28], warranting further investigation in ACB men.

## Conclusions

This community-based study identified challenges with PrEP delivery and uptake among ACB men, the majority of whom reported that they were unlikely to use PrEP. ACB MSW were much less likely to accept PrEP than ACB MSM. Self-perceived risk of acquiring HIV significantly impacted willingness of MSW to use PrEP, but was systematically under-estimated by participants. Taken together, this study informs the development of public health program strategies for PrEP scale-up among ACB men and identifies the need to develop targeted screening tools for these high-risk communities.

## Supporting information

S1 Table Questions used to assess participants’ knowledge about HIV and other STIs. (PDF)
