# Supplementary material for: Barriers to HIV pre-exposure prophylaxis among African, Caribbean and Black men in Toronto, Canada
Source: PLoS One. 2019 Mar 29;14(3):e0213740. doi: 10.1371/journal.pone.0213740 (PMC6440797; doi:10.1371/journal.pone.0213740)
Supplement: S1 Table — (PDF) [file pone.0213740.s001.pdf]

S1 Table. Questions used to assess participants' knowledge about HIV and other STIs.

1. People can protect themselves from HIV, the virus that causes AIDS, by using a condom correctly every time they have anal sex.
2. Having sex with only one faithful, uninfected partner can reduce the risk of getting or giving HIV.
3. A healthy looking person can have HIV.
4. When one partner is HIV-infected and has an undetectable viral load, it is less important that a condom be used.
5. Because there are now good HIV medications available, I am not very concerned about getting HIV.
6. With receptive oral sex (sucking your partner) without a condom, there is a small but significant risk of HIV infection.
7. If you have anal sex initially without a condom and then put one on before ejaculating, there is a significant risk of HIV infection.
8. I would always have symptoms if I was infected with HIV.
9. I would always have symptoms if I contracted a sexually transmitted infection.
10. Syphilis can be transmitted through unprotected oral sex.
11. I would always have symptoms if I was infected with hepatitis C.
12. There is treatment currently available for hepatitis C.
13. Condoms can effectively prevent sexually transmitted infections (STIs).
14. Condoms can effectively prevent HIV/AIDS.
15. You can use cooking oil or Vaseline for lubrication when using a condom.
16. Some space should be left at the tip of the condom to allow for sperm.
17. A condom should be unrolled before putting it on the penis.
